# Iodine: Its Role in Thyroid Hormone Biosynthesis and Beyond

**DOI:** 10.3390/nu13124469

**Published:** 2021-12-14

**Authors:** Salvatore Sorrenti, Enke Baldini, Daniele Pironi, Augusto Lauro, Valerio D’Orazi, Francesco Tartaglia, Domenico Tripodi, Eleonora Lori, Federica Gagliardi, Marianna Praticò, Giulio Illuminati, Vito D’Andrea, Piergaspare Palumbo, Salvatore Ulisse

**Affiliations:** Department of Surgical Sciences, “Sapienza” University of Rome, 00161 Rome, Italy; salvatore.sorrenti@uniroma1.it (S.S.); enke.baldini@uniroma1.it (E.B.); daniele.pironi@uniroma1.it (D.P.); augusto.lauro@uniroma1.it (A.L.); valerio.dorazi@uniroma1.it (V.D.); francesco.tartaglia@uniroma1.it (F.T.); domenico.tripodi@uniroma1.it (D.T.); eleonora.lori@uniroma1.it (E.L.); federica.gagliardi@uniroma1.it (F.G.); marianna.pratico@uniroma1.it (M.P.); giulio.illuminati@uniroma1.it (G.I.); vito.dandrea@uniroma1.it (V.D.); piergaspare.palumbo@uniroma1.it (P.P.)

**Keywords:** iodine, thyroid hormone biosynthesis, pregnancy, iodine prophylaxis, peroxidase, cancer, antioxidants

## Abstract

The present review deals with the functional roles of iodine and its metabolism. The main biological function of iodine concerns its role in the biosynthesis of thyroid hormones (THs) by the thyroid gland. In addition, however, further biological roles of iodine have emerged. Precisely, due to its significant action as scavenger of reactive oxygen species (ROS), iodine is thought to represent one of the oldest antioxidants in living organisms. Moreover, iodine oxidation to hypoiodite (IO^−^) has been shown to possess strong bactericidal as well as antiviral and antifungal activity. Finally, and importantly, iodine has been demonstrated to exert antineoplastic effects in human cancer cell lines. Thus, iodine, through the action of different tissue-specific peroxidases, may serve different evolutionarily conserved physiological functions that, beyond TH biosynthesis, encompass antioxidant activity and defense against pathogens and cancer progression.

## 1. Introduction

The term iodine comes from the French word “iode” originally proposed by J.P. Gay-Lussac, derived from the Greek “ἰοειδής” due to its characteristic violet color in its gaseous state [1]. Historically, the only biological function attributed to iodine concerned its incorporation into thyroid hormones (THs), synthesized by the thyroid gland [1]. THs, namely T_4_ (3,5,3′,5′-tetraiodo-L-thyronine) and T_3_ (3,5,3′-triiodo-L-thyronine), are characterized by the presence of four and three iodine atoms within the molecule, respectively, and play a prominent role in human body development and homeostasis [2,3]. In addition, over the last two decades, additional physiological roles of iodine have emerged [4,5,6,7]. Specifically, iodine is thought to represent one of the oldest terrestrial antioxidants used by living organisms due to its significant activity as a scavenger of reactive oxygen species (ROS) [5]. Moreover, iodine oxidation to hypoiodite (IO^−^) possesses strong bactericidal as well as antiviral and antifungal activity [6,7,8,9]. Finally, iodine has been demonstrated to exert antineoplastic effects in breast cancer, and in human melanoma- and lung cancer-derived cell lines [4,10,11,12,13,14]. Hereafter we will summarize the role of iodine in TH biosynthesis and define the detrimental consequences of iodine deficiency on human health [15,16]. We will then discuss the experimental evidence indicating a protective role of iodine against pathogens and cancer progression.

## 2. Sources of Dietary Iodine

Iodine content in food is highly variable, and is low in the majority of food and beverages. In drinking water, the iodine quantity is affected by a number of factors, including the iodine abundance in the soil, vicinity to seawater, and agricultural runoff [17]. In China, for example, the iodine concentration in water may be relatively high, ensuring an adequate or even excessive iodine intake, whereas in countries such as Israel that use desalinated water, the iodine amount is very poor [17,18,19]. In vegetables and fruits, the iodine quantity is mostly influenced by its presence in the soil, as well as by iodine-containing compounds employed in irrigation and fertilizers, and may vary from 10 μg per kg for plants grown on iodine deficient soils to 1 mg per kg for plants grown on iodine sufficient soils [1,17,20]. This in turn affects the dietary iodine intake of beef cattle, ovine animals, and poultry, together with iodine-enriched supplements, which are used in animal feed and salt licks [20]. Hens’ eggs may represent a source of iodine as they may contain between 23 and 43 μg of iodine per 100 g [21].

During lactation, the mammary gland produces milk with an iodine concentration that is 20–50 times greater than that of plasma, in order to meet the physiological iodine needs of the newborn. Therefore, milk usually contains a discrete amount of this element, which may vary from 33 to 534 μg/L [20,22,23]. The latter amounts could be also influenced by iodine-containing disinfectants used in milk production, such as iodophors, which are employed to clean milk containers and udders [20,22,23]. It has been estimated that milk and dairy products may contribute 13–64% of the recommended daily iodine intake [23].

The highest iodine content is found in fish and marine plants, which are capable of accumulate it from seawater [1]. The iodine concentrations in seawater fish species vary between 18 μg and 1210 μg/100 g [1,21,24,25]. Not surprisingly, the iodine content in freshwater fishes is about six times lower than that in marine fishes, though they may have overlapping ranges of values [24]. The iodine content of macroalgae (seaweed) is also very variable and is found to range from 16 μg/g to over 8165 μg/g [25,26,27]. Seaweed consumption, originally limited to Asian countries, has now entered the global food market, representing a new source of iodine intake for Western populations [28]. It is worth mentioning, however, that eating too much seaweed that is rich in iodine could be detrimental to thyroid function [29]. Indeed, excessive iodine consumption from seaweed has been associated with thyroid disorders and may have adverse effects on susceptible population groups, such as pregnant women and subjects affected by thyroid autoimmunity [30,31,32,33,34,35,36].

In many countries, where an iodoprophylaxis program has been adopted, an important source of iodine is represented by the use of iodized salt in both households and the food industry. An additional source is dietary supplements containing iodine. In the United States, data from the third National Health and Nutrition Examination Survey (NHANES III) indicated that about 15% of the adult population consumed iodine-containing supplements, which provided about 140 μg/day of iodine [1,37].

## 3. Iodine Metabolism

Iodine is mainly ingested as iodide (I^−^); iodate (IO_3_^−^), which is generally used in salt iodization; or organically bound iodine. More than 90% of ingested iodide is absorbed in the duodenum [1,4,38]. Iodate is reduced in the gut to iodide before absorption, whereas the organically bound iodine is digested, and the released iodide is absorbed [1]. The uptake is mediated by the sodium/iodide symporter (NIS), present on the apical plasma membrane of enterocytes of the duodenum, jejunum, and ileum [39,40,41]. Moreover, other carriers expressed on the brush border of enterocytes are thought to contribute to iodide absorption in the gut, including the sodium multivitamin transporter (SMVT) and the cystic fibrosis transmembrane conductance regulator (CFTR) [4,42,43]. Once absorbed, iodide is transferred to the bloodstream through molecular mechanisms that are still to be fully clarified [4]. In addition to intestinal absorption, deiodination of T4 and T3 by deiodinases in peripheral tissues contributes to the level of iodide present in the bloodstream [44]. Circulating iodide is either taken up by the thyroid gland through the action of NIS, present in the basolateral plasma membrane of thyrocytes, or eliminated in the urine. Inside the kidney, NIS expression was first localized by means of immunohistochemistry in the basolateral membrane of distal tubular cells, suggesting their role in iodide excretion [45]. However, different results were obtained in subsequent studies in which NIS expression was observed on the apical membrane of cells belonging to proximal and cortical collecting tubes, indicating a resorption action of iodide from the urine [4,46]. Thus, the urinary excretion of iodine could be the result of these two opposite processes. It is worth mentioning that in conditions of adequate dietary iodine intake, no more than 10% of iodine absorbed in the gut is retained by the thyroid gland, whereas in conditions of chronic iodine deficiency the thyroid gland may catch more than 80% of the bloodstream iodide [1,47].

During gestation and lactation, circulating iodide is also cleared by the fetal thyroid, which starts to be functional from the middle of the 2nd trimester of gestation onward, and during lactation by the mammary gland, which concentrates iodide in milk to ensure correct thyroid functioning in the newborn [48,49,50,51]. The maternal transfer of iodine to the fetus is ensured by the expression of NIS and PENDRIN, a sodium-independent chloride-iodide exchanger, within the placenta [49]. The observation that the placental expression of NIS is already high at 8–10 weeks of gestation, when the fetal thyroid is not yet ready to synthesize THs, suggests that iodide may have additional functional roles during this period (i.e., antioxidant activity and defense against infections) [4]. In the mammary gland, the iodide concentration is ensured by the expression of NIS in the basolateral membrane of mammary alveolar cells [4]. Secretion in milk is then performed by the CFTR, anoctamin-1 (ANO1), and PENDRIN iodide transporters, localized in the apical side of the cell membrane [4].

## 4. Iodine Metabolism in the Thyroid

As mentioned, the main physiological function of iodine concerns TH biosynthesis by the thyroid gland [1]. Bloodstream iodide is actively transported across the plasma membrane into the cytoplasm of thyrocytes by NIS, exploiting the concentration gradient of Na^+^ generated by the Na^+^/K^+^-ATPase transporter as a driving force [46,52]. Iodide is then transferred to the lumen of thyroid follicles by several transporters, including PENDRIN, ANO1, and CFTR [53,54,55,56]. Here, at the outer surface of the apical membranes of thyrocytes, the biosynthesis of THs is initiated by thyroid peroxidase (TPO), which uses H_2_O_2_ produced by DUOX2 to oxidize iodide to iodine radicals and incorporates it on specific tyrosine residues within thyrocyte-secreted thyroglobulin (Tg) molecules [57]. After that, TPO couples two residues of diiodotyrosine (DIT) to form thyroxine (T_4_), and one monoiodotyrosine (MIT) to one DIT to form thyroxine (T_3_). Mature Tg, containing THs, is stored in the colloid of the follicular lumen. The secretion of THs relies on Tg reabsorption from the lumen by micropinocytosis, and its proteolysis by lysosomal enzymes that release THs from the Tg protein [57]. Uncoupled MIT or DIT residues are deiodinated by the iodotyrosine dehalogenase (DEHAL1), a transmembrane protein localized mainly at the apical pole of thyrocytes and involved in the intrathyroidal recycling of iodide [58]. THs are transported outside the basolateral membrane of thyrocytes, mainly by monocarboxylate transporter 8 (MCT8), from which they reach the bloodstream. The pituitary thyroid stimulating hormone (TSH), through its receptor (TSHR) present on the basolateral surfaces of thyrocytes, is the mainly regulator of TH biosynthesis, controlling the expression of the thyroid-specific genes involved in TH biosynthesis [4,57].

## 5. Other Micronutrients and Goitrogens

Deficiencies of other micronutrients, such as selenium and iron, required for the optimal function of key enzymes involved in TH biosynthesis, can lead to decreased TH production, exacerbating the effects of iodine deficiency [59]. Observations have shown that iron supplementation may improve the efficacy of iodine supplementation in iodine- and iron-deficient children [60,61].

It is also worth considering that a number of compounds naturally contained in foods such as sorghum, soy, millet, and cassava, or pollutants found in food and water (i.e., perchlorate and nitrate) may negatively affect iodine metabolism and thyroid function [62]. These substances, known as goitrogens, can either compete with iodine uptake by thyrocytes or impair the activity of enzymes essential for TH biosynthesis. Newborns and children seem to be particularly sensitive to goitrogens, which could worsen pre-existing iodine deficiency.

## 6. Recommended Daily Iodine Intake and Its Assessment

The iodine requirement in humans varies with age [1]. The daily iodine intake, recommended by the United Nations Children’s Fund (UNICEF), the International Council for Control of Iodine Deficiency Disorders (ICCIDD), and the World Health Organization (WHO) for different age groups, is as follows: preschool children (0 to 59 months)—90 μg/day; schoolchildren (6 to 12 years)—120 μg/day; adults (above 12 years)—150 μg/day; pregnant and lactating women—250 μg/day [15,16]. Note that the daily requirement of iodine in pregnancy has increased from 150 μg/day to 220–250 μg/day. This surplus is needed to (i) satisfy the greater maternal TH production required to guarantee maternal euthyroidism and the transfer of THs to the fetus before the fetal thyroid starts to function; (ii) provide the iodine necessary for TH biosynthesis by the fetal thyroid in the second and third trimesters of pregnancy; (iii) balance the augmented maternal renal iodine clearance; (iv) compensate for the increased degradation of T_4_ to reverse T_3_ due to the expression of type 3 deiodinase in the placenta [63,64]. The daily iodine needs remain elevated during lactation (250–290 μg/day) in order to guarantee the correct amount (approximately 115–150 μg/day) of iodine in the milk of lactating women [37,65]. As stated above, in conditions of adequate dietary iodine intake, no more than 10% of iodine absorbed in the gut is taken up by the thyroid gland, with the majority of remaining iodine (>90%) excreted in the urine, whereas the percentage eliminated in the feces is minimal [1,15]. For this reason, the urinary iodine concentration (UIC), expressed in μg/L and obtained from spot urine specimens, is the best indicator used to evaluate the median iodine intake in a given population [1,15]. Assuming a median 24 h urine volume of 1.5 L, a UIC value of 100 μg/L corresponds to a daily iodine consumption of 150 μg. Table 1 and Table 2 show the median UICs and related levels of iodine assumption in a given population and in pregnant women, respectively, as estimated by the WHO [15,16]. The median UIC in urine sample spots from an adequate number of school-age children (SAC) is considered a trustworthy marker for iodine intake by the general population of a given area [15].

It has to be taken into account, however, that UIC determined on urine sample spots is unreliable when measuring iodine intake in single individuals, owing to day-to-day variability in feed and hydration levels [1,66,67,68]. In that case, UIC determination on several urine spots collected within 24 h should be performed, but this is rather unfeasible. Alternative methods have been proposed, for example, using the age- and sex-adjusted iodine/creatinine ratio in adult individuals, although these have major limitations [1].

An additional feasible approach to assess iodine intake in a given population is through the prevalence of goiter estimated through ultrasonography [69,70,71,72]. In Table 3, the correspondence between goiter prevalence and iodine nutritional status in a given population is reported.

## 7. Consequences of Iodine Deficiency

Iodine deficiency has several detrimental effects on human growth and development [1,15,16]. About four decades ago, Basil S. Hetzel first coined the term “iodine deficiency disorders” (IDD), recognizing how the negative consequences of a poor dietary iodine intake extended far beyond simple goiter [73,74,75]. As summarized in Table 4, the health risks associated with iodine deficiency may persist throughout the lifetime.

As can be seen in Table 4, hypothyroidism consequent to iodine deficiency in women causes important reproductive alterations including anovulation and reduced fertility and, when pregnancy occurs, gestational hypertension, stillbirths, and congenital anomalies, and increased perinatal mortality may be observed [76]. This may have cultural and socioeconomic consequences, compromising the life quality of parents that face the responsibility of taking care of a child with serious health problems [76,77]. From a physiological point of view, infertility occurring in iodine-deficient hypothyroid women could be seen as a protective mechanism implemented by the body to avoid hazards related to pregnancies carried out in iodine deficiency conditions. Recently, data indicating a direct association between iodine status and fertility have been reported by Mills and colleagues [78]. They observed that the time to pregnancy was significantly delayed in women with preconception UIC values <50 μg/L, with a fecundability odds ratio reduced by 46% over each menstrual cycle [78]. The detrimental effects of iodine deficiency on the development and maturation of the fetal brain are of particular relevance, representing a major preventable cause of mental defects [1,15,16,79,80,81,82,83,84,85,86]. In fact, appropriate TH levels are essential to neural migration and brain myelinization from the fetal to the early postnatal period [87,88,89,90]. Hypothyroxinemia during this critical window induces irreversible brain damage, leading to mental retardation and neurological abnormalities [1,15,16,79,80,81,82,83,84,85,86]. Infant cretinism is well known to be associated with endemic goiter, but the existence of a continuum from mild mental retardation to gross neurological impairment takes place depending on the level of iodine insufficiency [79,80,81,82,83,84,85,86]. It has been suggested that iodine deficiency results in a loss of 13.5 intelligence quotient (IQ) points at the level of the global population [87]. This could have negative effects on socioeconomic development, noticably reducing the gross domestic product of a given population, and is why brain damage and the loss of intellectual potential, in addition to endemic goiter, should be considered a major public health problem, especially in developing countries [78,87,88].

## 8. Iodine Functions against Pathogens

In the salivary glands, stomach, and intestine, iodide is thought to take part in innate immune defense [4]. In these tissues, iodide may be recycled from the bloodstream and eventually re-absorbed again by the epithelial cells of the duodenum, jejunum, and ileum [4]. In the salivary glands and in the mucin-secreting and parietal cells of the stomach, iodide uptake from the bloodstream is mediated by the NIS present on the basolateral membrane of the cells, and secreted in saliva and gastric juices by low-affinity iodide transporters on the apical plasma membranes, such as CFTR, ANO1, and PENDRIN [4,8,89,90,91,92,93,94]. The presence of DUOX2 in the apical membrane of epithelial salivary cells, of the gastric mucosa, and of the apical surface of enterocytes, along with that of tissue-specific peroxidase (salivary peroxidase, gastric peroxidase, and lactoperoxidase (LPO) in the intestinal mucus) in these sites allow for iodide oxidation to hypoiodite (IO^−^), which is endowed with fungicidal and bactericidal activity [4,6,7,8,89,90,91,92,93,94]. In addition, through a similar mechanism to that described above, iodide oxidation has been shown to possess a strong antiviral action against lung adenoviruses [4,9]. On these bases, a placebo-controlled trial aiming to measure the efficacy of the iodide treatment of patients with mild to moderate COVID-19 in Pakistan was initiated in July 2020 and ended in August 2021 [95]. The results, however, are still to be communicated.

## 9. Iodine and Cancer

Several experimental findings have indicated that iodide may elicit antiproliferative and apoptotic effects in malignant cells [4]. Vitale and colleagues reported the ability of an excess of iodide (KI) to induce apoptosis of immortalized thyroid cells and primary cultures of human thyrocytes, but not of extrathyroidal cells [96]. The apoptotic effect of thyroid cells was p53-independent and required the presence of a functional thyroid peroxidase as its inhibition by propylthiouracil completely prevented iodide-induced apoptosis [9]. Later on, Zhang and colleagues confirmed the ability of iodide to induce apoptosis of lung cancer cells transfected with NIS and TPO, but not in those transfected only with NIS [97]. Altogether, this evidence indicates that iodide needs to be oxidized to induce apoptosis. García-Solís and colleagues analyzed the effect of molecular iodine (I_2_) and iodide (KI) on the induction and promotion of mammary cancer induced by N-Methyl-N-nitrosourea in rats [98]. They found that I_2_, but not iodide, had a potent antineoplastic effect on the progression of mammary cancer. This antineoplastic effect was thought to be mediated by I_2_-induced expression of the peroxisome proliferator-activated receptor γ (PPARγ), which is capable of triggering the apoptosis of malignant cells [98,99,100]. The observation that iodide had no effects in this experimental system was explained by the lack or low expression of NIS and/or LPO. In a different human mammary cancer model, using dimethylbenz[a]anthracene (DMBA)-induced mammary tumors, the tumor cells were shown to express both NIS and LPO [101]. In this experimental model the co-administration of iodine and/or iodide along with medroxyprogesterone acetate (MPA) inhibited mammary cancer growth at a significantly higher level with respect to MPA alone [102,103]. In particular, the higher growth inhibitory effects were observed in tumor tissues with a higher iodine content, indicating that direct iodine uptake by breast tumors led to the suppression of tumor growth [102]. More recently, iodine supplementation was found to enhance the antineoplastic effect of doxorubicin in canine patients affected by mammary cancers [13,14]. In particular, co-treatment with doxorubicin and I_2_ has been shown to improve therapeutic outcomes, diminish the invasive capacity, attenuate adverse events, and increase disease-free survival. The antiproliferative and apoptotic effects of iodine were further confirmed in thymic epithelial tumour cells and different human colon cancer cell lines [11,12]. Altogether, this experimental evidence clearly suggests that iodine may positively contribute to the fight against cancer.

## 10. Conclusions

Iodine is an essential micronutrient required for proper TH biosynthesis that plays critical roles in development, growth, and metabolism. The lack of an adequate iodine intake with the diet, and a consequent reduction of TH levels, may have detrimental health consequences, generating a number of lifetime disorders known as iodine deficiency disorders. Over the last couple of decades, it has become clear that the functional roles of iodine extend beyond that of TH biosynthesis, as it has roles in the innate immune response against pathogens and as an anticancer agent, which warrant further investigations.

## Figures and Tables

**Table 1:** Assessment of iodine intake in the general population based on median urinary iodine concentrations (UICs). Adapted from [15,16].

Median UIC	Iodine Intake	Nutritional Status
<20 μg/L	Insufficient	Severe iodine deficiency
20–49 μg/L	Insufficient	Moderate iodine deficiency
50–99 μg/L	Insufficient	Mild iodine deficiency
100–299 μg/L	Adequate	Optimal
≥300 μg/L	Excessive	Risk of adverse health consequences (iodine induced hyperthyroidism, autoimmune thyroid diseases)

**Table 2:** Assessment of iodine assumption in pregnant women based on median urinary iodine concentrations (UICs). Adapted from [16].

Median UIC	Iodine Intake
<150 μg/L	Insufficient
150–249 μg/L	Adequate
250–499 μg/L	More than adequate
≥500 μg/L	No added health benefit is expected

**Table 3:** Assessment of iodine intake in the general population based on goiter prevalence. Adapted from [16].

Goiter Prevalence	Nutritional Status
<5%	Iodine sufficiency
5.0%–19.9%	Mild iodine deficiency
20.0%–29.9%	Moderate iodine deficiency
>30%	Severe iodine deficiency

**Table 4:** Iodine deficiency disorders at different ages. Adapted from [1,71,76].

Age	Iodine Deficiency Disorders
Fetus	Abortions, stillbirths, congenital anomalies Increased perinatal mortality
Neonate	Neonatal hypothyroidism, endemic cretinism Increased susceptibility of the thyroid gland to nuclear radiation
Child and adolescent	Goiter, hypothyroidism or hyperthyroidism Impaired mental function, delayed growth and puberty Increased susceptibility of the thyroid gland to nuclear radiation
Adult	Goiter with its complications, hypothyroidism Infertility, Impaired mental function Spontaneous hyperthyroidism in the elderly Iodine-induced hyperthyroidism Increased susceptibility of the thyroid gland to nuclear radiation

## Data Availability

Not applicable.
